# In situ observation of biotite (001) surface dissolution at pH 1 and 9.5 by advanced optical microscopy

**DOI:** 10.3762/bjnano.6.67

**Published:** 2015-03-05

**Authors:** Chiara Cappelli, Daniel Lamarca-Irisarri, Jordi Camas, F Javier Huertas, Alexander E S Van Driessche

**Affiliations:** 1 Instituto Andaluz de Ciencias de la Tierra (IACT - CSIC-Universidad de Granada), Avda. de las Palmeras 4, 18100 Armilla, Granada, Spain; 2 Institute of Environmental Assessment and Water Research (IDAEA), CSIC, Jordi Girona 18–26, 08034 Barcelona, Spain; 3 Structural Biology Brussel, VUB, Pleinlaan 2, 1050 Brussels, Belgium

**Keywords:** biotite, dissolution mechanism, environmental, in situ observation, pH effect

## Abstract

Laser confocal differential interference contrast microscopy (LCM-DIM) allows for the study of the reactivity of surface minerals with slow dissolution rates (e.g., phyllosilicates). With this technique, it is possible to carry out in situ inspection of the reacting surface in a broad range of pH, ionic strength and temperature providing useful information to help unravel the dissolution mechanisms of phyllosilicates. In this work, LCM-DIM was used to study the mechanisms controlling the biotite (001) surface dissolution at pH 1 (11 and 25 °C) and pH 9.5 (50 °C). Step edges are the preferential sites of dissolution and lead to step retreat, regardless of the solution pH. At pH 1, layer swelling and peeling takes place, whereas at pH 9.5 fibrous structures (streaks) form at the step edges. Confocal Raman spectroscopy characterization of the reacted surface could not confirm if the formation of a secondary phase was responsible for the presence of these structures.

## Introduction

The study of the reactivity of silicate minerals is essential to understand numerous bio-geochemical processes. Silicate weathering plays an important role in the carbon cycle, the formation of soil and the nutrition of plants [1]. Moreover, the release of cations from silicates and the high cation-exchange capacity of some phyllosilicates contribute to the pH stability of natural waters, the mobility of metals and the control of potentially toxic elements [2–3].

Flow-through reactors filled with powdered samples are frequently used to study the reaction mechanisms of mica dissolution and possible formation of new phases [3–9]. In this type of experiment, the full control over the parameters that influence the reactions (e.g., flow rate, pH, temperature and solution composition) allows one to quantify the mineral dissolution rates and the study of the reaction mechanisms under a wide range of experimental conditions. However, this experimental approach is rather unapt to deal with the reactivity of each crystal face, elucidate the face-specific dissolution–precipitation mechanisms and determine the specific location of the secondary mineral formation. In the last decades, the use of several advanced microscope techniques has allowed for the inspection of the mineral surfaces with high spatial resolution to explore morphological and topographical changes during the alteration process. Atomic force microscopy (AFM) is often employed to characterize reactive surface areas of silicate minerals in situ. For example, dissolution features and precipitation phases can be identified for a field of view that ranges from hundreds of nanometers to 120 micrometers with Angstrom resolution in the vertical plane [1,10–21]. Likewise, ex situ observations of micro-topographic changes on silicate surfaces over larger fields of view (90–2000 µm) are possible with nanometer-scale vertical resolution by using vertical scanning interferometry (VSI) [22–25]. Recently, Tsukamoto and coworkers designed a high-resolution phase shifting interferometer (PSI) that allows for the in situ measurement of extremely low surface dissolution (and growth) rates of minerals while submerged in aqueous solutions [26–30]. With the progress of these techniques our understanding of the mechanisms of the surface reactivity of phyllosilicates has greatly improved.

Although great progress at the experimental and theoretical front has been achieved, further investigations are needed to determine the precise mechanisms of phyllosilicate weathering (especially for low-reactivity conditions) and to integrate them with the new insights of theoretical models developed in the last decade. The main goal of the present work is to show the capability of the confocal differential interference contrast microscopy (LCM-DIM) to study phyllosilicate dissolution in situ. As mentioned above the capability of the AFM and VSI techniques to study mineral reactivity is remarkable, but each one alone shows some limitations [31]. AFM allows for the high-resolution characterization of surface features at the monolayer range but over narrow fields of view, preventing to investigate surface phenomena at the mesoscale. In the case of VSI, the field of view is wider and long in situ observations are possible. However, measurements are highly sensitive to small fluctuations of temperature and air bubbles. Instead, in situ measurements under different solution pH, temperature, flow rate, and pressure by using flow-through cells can be performed with LCM-DIM with a vertical resolution of about 1 nm over a wide field of view (ca. 0.3–2 mm). Although it only provides qualitative height information [31], morphological changes on mineral surfaces are suitably monitored. Additionally, owing to the relatively fast data acquisition (ca. 9.6 s to scan an area of 800 × 800 μm^2^ [31]) and acquired stability, LCM-DIM allows for a stable surface monitoring over long time spans (up to months). AFM, VSI/PSI and LCM-DIM techniques are therefore complementary, and with the latter technique precise information of surface reactivity of slow dissolving minerals at the micro- and meso-scales over long time can be obtained.

In this study we investigate the reactivity of the cleaved biotite (001) surface, at pH 1 and pH ca. 9.5, by using in situ flow-through LCM-DIM experiments, combined with phase shifting interferometry (PSI). The experimental results are discussed considering the most relevant theories on mineral/solution interface processes, i.e., step wave model, dissolution/re-precipitation and leached layer mechanisms [22,32–37].

## Results and Discussion

**Figure 1:**
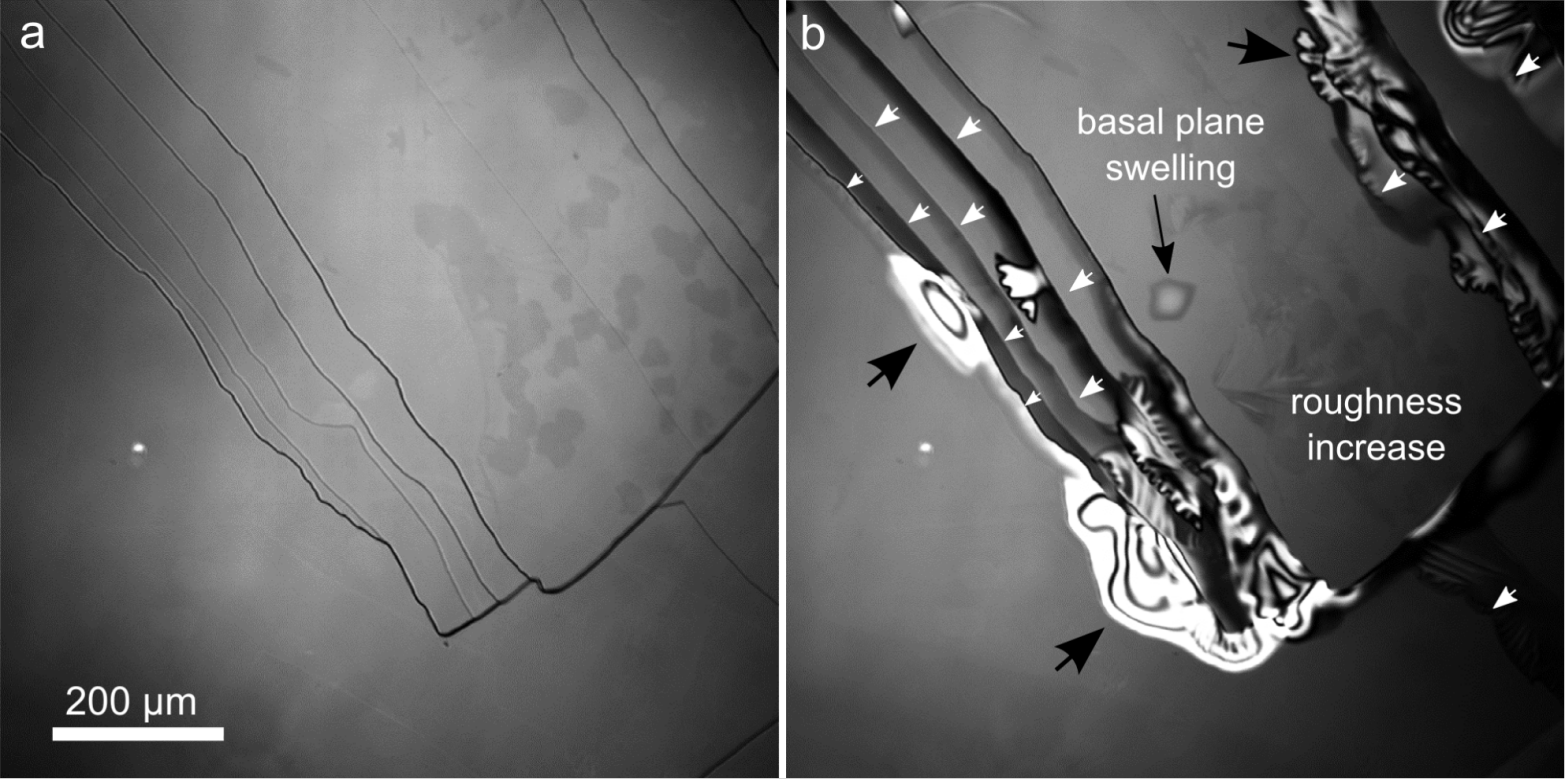
LCM-DIM images of a) freshly cleaved biotite (001) surface and b) the same surface after about 17 h of reaction at pH 1 and 25.0 °C. Upper layers appear in some areas “overexposed” (white zones) owing to layer breaking and curling (peeling process) after swelling. Black arrows indicate areas with layer swelling and peeling, and white arrows indicate the dissolution direction (see text).

Figure 1a shows LCM-DIM images of a freshly cleaved biotite (001) surface with visible terrace limits. The darker the outline (i.e., contrast), the higher the step. The same surface reacted for ca. 17 h at pH 1 and 25 °C shows edge retreat, layer swelling and peeling (Figure 1b), the latter processes being a consequence of biotite dissolution.

Aldushin et al. [10] suggested that the reaction front on the phlogopite surface was caused by the exchange of interlayer K^+^ ions, by octylammonium ions and reported a retreat rate of about 4 × 10^−4^ µm/s at the initial stage, which decreased to 1 × 10^−4^ µm/s and about 3 × 10^−5^ µm/s for phlogopite dissolution at 20 °C and pH 7. Cappelli et al. [38] reported rates of 7.5 × 10^−4^ and 3.5 × 10^−3^ µm/s for biotite (001) surface retreat of low steps at 11.5 and 25 °C and pH 1, respectively. Although the rates of Aldushin et al. at pH 7 are slower than the rates at pH 1 measured by Cappelli et al., it is insufficient to discard that an exchange between Na^+^ and K^+^ is not involved in the fast edge retreat observed on the biotite (001) surface, similarly to that reported by Sánchez-Pastor et al. [21] for phlogopite. However, additional interferometry observations of biotite surfaces reacted with inorganic and organic acids over a wide temperature range (data not shown, in preparation) point to a retreat of low steps due to dissolution rather than ion exchange.

**Figure 2:**
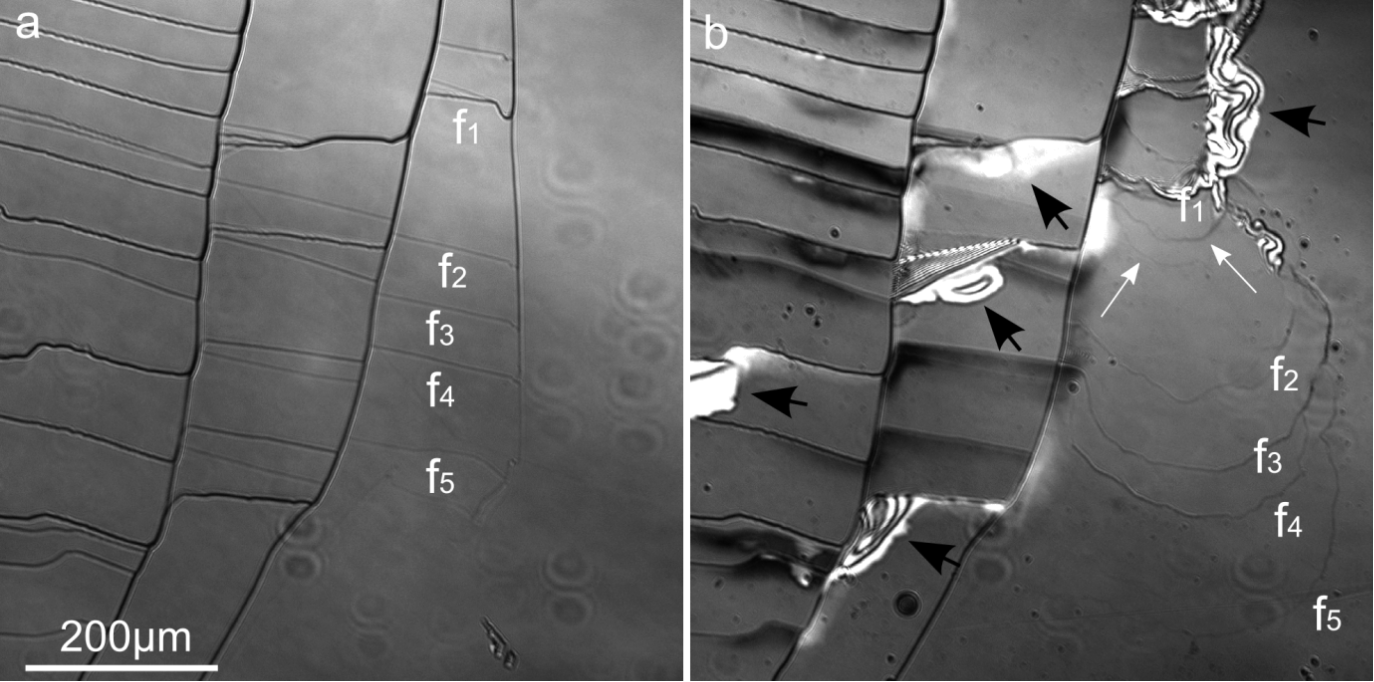
LCM-DIM images: a) freshly cleaved biotite (001) surface and b) the same surface after about 63 h at pH 1 and at 11.5 °C. Black arrows indicate swelling and peeling layers. White arrows indicate new dissolution fronts breaking away from slower moving step edges. Darker areas in b) correspond to higher steps whose upper layers are dissolving. Lower steps (f_1_–f_5_) move across the surface following a semi-circular pattern and creating unaltered biotite surface. The pale elliptic structures are dust particles derived from the objective lens.

In the case of macrosteps, monolayers or bunches of layers spread while the position of the macrostep remains apparently unchanged (Figure 1). This peculiar behavior is related to the presence of steps with different height on the basal surface. As described in Cappelli et al. [38], while low steps clearly retreat, leaving fresh unaltered surface, the position of high steps does not change, as only dissolution of the upper layers occurs. Indeed a series of time-lapsed LCM-DIM images (Figure 2a) shows that only upper layers dissolve from high steps while these macrosteps do not lose their initial position (darker areas, Figure 2b) and low steps move across the surface creating fresh biotite surface. Dissolution fronts (f_1_–f_5_) propagate following a semicircular pattern, indicating that the dissolution rate is similar in all crystallographic directions. Interestingly, new dissolution fronts break away from slower moving step edges (white arrows, Figure 2b), indicating that upper layers move faster than lower ones. Basal plane swelling and a general increase of roughness were also observed.

Figure 1 and Figure 2 show that step edges do not dissolve uniformly except in the case of low steps. Cappelli et al. [38] observed that the retreat rate changes with the step thickness, being higher for low steps. Likewise, in this study, it is observed that the dissolution started at specific locations. These observations are in accordance with the results of recent studies that argue about the validity of an average dissolution rate value for complex minerals [32–33]. The novel concept of a rate spectrum was introduced for the complex anisotropic dissolution of mineral surfaces; this implies the existence of a surface energy distribution. In agreement with the above consideration the variability of biotite reactivity is an intrinsic factor of its crystalline anisotropy, i.e., surface energy variance, and thermodynamic parameters, such as activation energy, are not representative of the overall mineral dissolution process. For this reason the activation energy value reported in Cappelli et al. [38] would be part of a probability distribution and could only be associated to the low step retreat.

In the same way biotite step-edge alteration can be reviewed based on the theory of dissolution/re-precipitation for silicate weathering [35–36,39–40]. Based on the results of other studies [11,20,41–43], in our previous work [38] we proposed that layer swelling and peeling likely occurred in three consecutive steps: (1) initial leaching of interlayer and octahedral cations from the biotite structure; (2) hydrolysis of the Si–O–Si and Si–O–Al groups of the tetrahedral sheet, responsible for the layer expansion and (3) re-polymerization of Si–OH groups to form Si–O–Si that might cause layer contraction by expulsion of water. In a new concept of the mineral/solution interface processes [36] the formation of the so called leached layer, due to the loss of octahedral and interlayer cations, is substituted by the existence of a dissolution/re-precipitation interface at which amorphous silica-rich surface layers form [40]. The always stoichiometric dissolution of the mineral is followed by the precipitation of a secondary phase in spite of an undersaturated bulk solution with respect to that secondary phase [36]. In agreement with this theory the increase of layer thickness could correspond to the newly formed silica layer. Yet, layer curling and peeling, observed also in previous studies [43–44], are not fully accounted for by this model.

**Figure 3:**
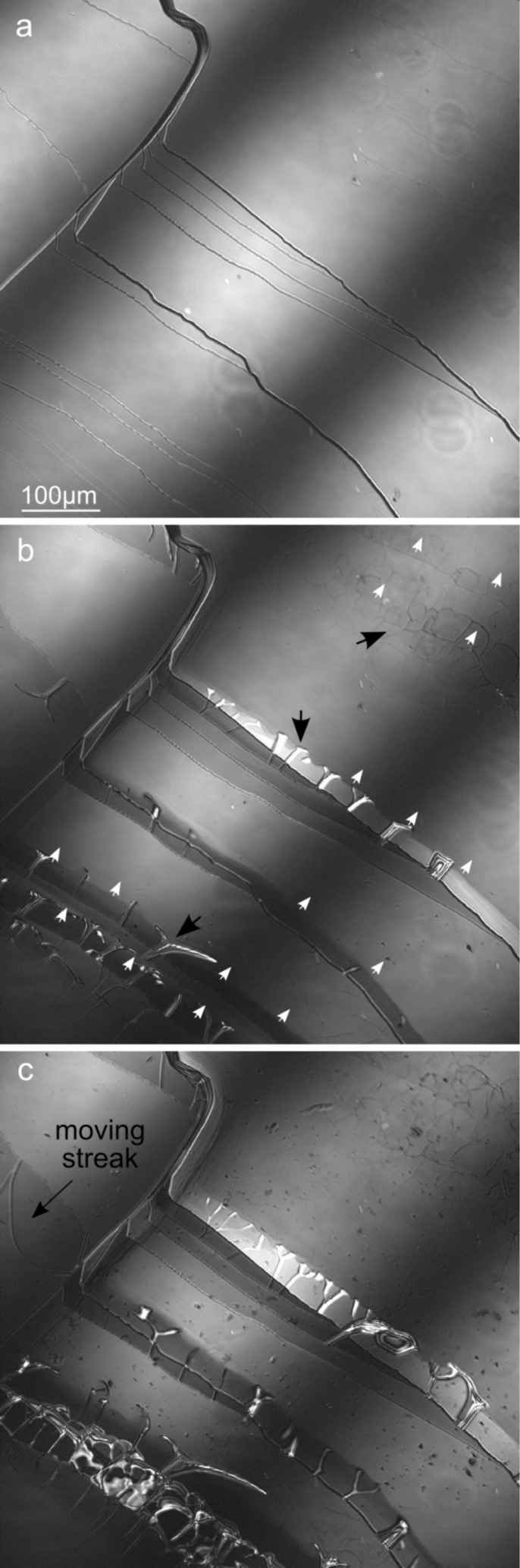
LCM-DIM images: a) freshly cleaved biotite (001) surface; b) same surface after about 33 h and c) after about 51 h at pH 9.5 and 50 °C. White arrows and black arrows in b) indicate edge retreat and streaks, respectively. Streaks formed (b) and grew (c) from steps edge.

**Figure 4:**
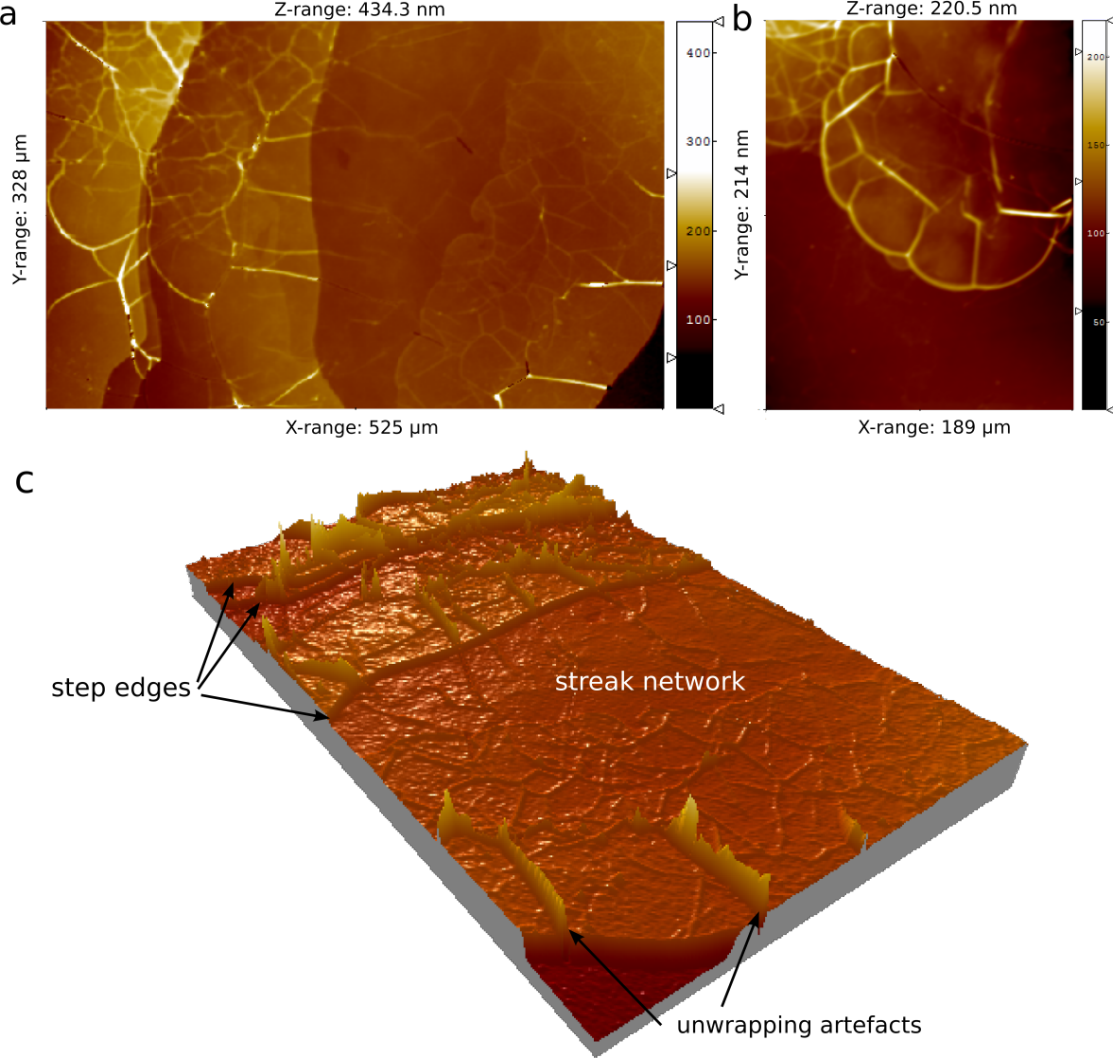
Unwrapped phase shift interferograms of a reacted biotite (001) surface at pH 9.5 and 50 °C after about 3 d: a) 2D image that shows high (80–100 nm) and low (8–10 nm) streaks, which spread from step edges; b) 2D image that shows low streaks in more detail; c) 3D image of a).

At basic pH, dissolution also occurred through edge retreat. However, while at acidic pH precipitation of new phases was not observed, at basic pH the dissolution of the biotite (001) surface produced new structures, namely streaks, that grew from step edges and were associated with precipitation (Figure 3). During the early stage of dissolution streaks developed close to steps edges, spreading thereafter over the entire (001) surface (Figure 4). Sánchez-Pastor et al. [21] reported the formation of streaks with heights of 200 nm on phlogopite surfaces during dissolution at room temperature. These streaks were described as irregular swelling structures (bulge-type shapes). Their formation was associated with an excess of water uptake influenced by local variations of the TOT-layer charge. Likewise, Aldushin et al. [10] observed bulge formation (with heights up to 50 nm) on phlogopite induced by octylammonium–K^+^ exchange, arguing that these swelling structures reorganized themselves in new configurations after some reaction time. A similar behaviour was observed in our experiments in which some streaks started to move after an induction time and changed their arrangement (Figure 3). The streaks developed as fiber-type structures with heights that ranged between 10 and 100 nm (measured by PSI with a Linnik configuration; Figure 4). Extensive evidence exists about mica transformation and formation of secondary phases during weathering over a wide range of experimental conditions [3,5–6,19,45–46]. Hu et al. [45] observed the formation of “fibrous illite structures” when biotite reacted in 1 mol·L^−1^ NaCl solution and high temperature (acidic hydrothermal conditions). Shao et al. [47] observed the formation of fibrous illite phases on reacted phlogopite surfaces in presence of organic acids under geologic CO_2_ sequestration conditions (95 °C and 102 bar). Based on AFM observations, the authors argued that nano-particles can migrate over mineral surfaces (in particular from edge to basal surface) [46].

**Figure 5:**
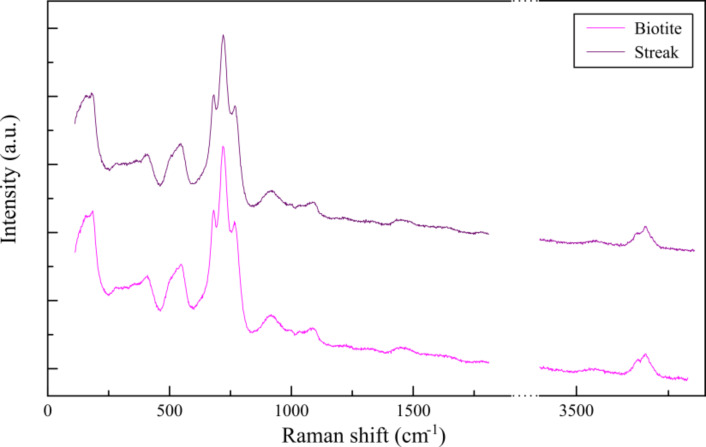
Comparison of characteristic Raman spectra of the (001) biotite surface and that of a streak formed at pH 9.5 and 50 °C. No differences between both spectra are detected.

To identify the nature of the streak structures, the reacted biotite surface was analysed by confocal Raman spectroscopy. The spectra of the unreacted (001) basal surface and that of the reacted surface with the streaks only showed biotite peaks (Figure 5). However, it should be noted that, owing to the penetration depth (ca. 100 µm) of Raman spectroscopy and the consequent strong “background” signal from the bulk biotite phase with respect to the weak signal from the secondary phase(s), we cannot confirm nor refute the presence of new mineral phase(s). In addition, the measured chemical composition of the output solution of an experiment conducted at pH 9.5 shows a deficit in aqueous Al and Fe, as well as a higher Mg concentration than that of Si (Table 1). The calculated saturation index (SI) values show that iron oxyhydroxide (goethite) or aluminum oxyhydroxide (boehmite), as well as some Mg-bearing aluminosilicate minerals (talc, phlogopite and saponite) could precipitate (SI > 0, Table 2).

**Table 1:** Cation concentrations (µmol·L^−1^) of the output solution at pH 9.5 and 50 °C (d.l.: detection limit).

element	run 1 µmol·L^−1^	run 2 µmol·L^−1^

Si	27.8	26.0
Al	<d.l.	1.5
Fe	4.5	3.6
Mg	16.9	16.5

**Table 2:** PHREEQC saturation indexes calculated with the measured composition of the output solution at pH 9.5 and 50 C.

phase	SI

boehmite	−2.94
brucite	2.81
Fe(OH)_3_	0.11
goethite	4.91
muscovite	−16.13
nontronite-Na	−0.64
phlogopite	7.56
quartz	−3.59
saponite-Na	7.34
sepiolite	−0.78
SiO_2_(am)	−4.71
talc	5.12

Although the derivation of the surface charge of multi-oxide silicates as a function of pH is complex and requires the knowledge of all zero point charge parameters (e.g., isoelectric point, point of zero net proton charge, point of zero salt effect) for an unambiguous description of biotite surface chemistry [48], in general, the alkali treatment of silicate mineral affects the variable surface charge in a way that reactivity towards charged and polar compounds should increase (increase in surface acidity) [49]. This could be responsible for the initial adsorption of particles along the edge surface, where a variable charge is present. Precipitates would then grow and expand on the biotite (001) surface forming a fiber-like structure. Similarly, Johnsson et al. [50] observed small fibrous structures by using AFM on muscovite basal surface after two days of reaction time at pH 5.7 at 22 °C. After ten days of reaction the fibers formed a network with a height of 8–12 Å, covering 20% of the sample surface.

Although the formation of oxides, hydroxides and aluminosilicate phases is likely to occur at the expense of biotite dissolution at basic pH, additional experiments are necessary to confirm or refute precipitation of secondary phases.

## Conclusion

In situ LCM-DIM inspection, of the reacted biotite (001) surfaces has shown the differences between the basal surface reactivity in acidic (pH 1) and basic (pH 9.5) solutions. In both pH values step edges are preferential sites of dissolution, leading to step retreat. Layer swelling and peeling occur in acidic pH, while at basic pH fibrous structures (streaks) formed at step edges, whose temporal evolution was monitored in situ by LCM-DIM. Precipitation appears to be responsible for the formation of streaks.

The experimental approach based on LCM-DIM is a promising technique to study in situ the surface alteration of mica (and other minerals) over a wide range of solution composition and temperature. The obtained (001) surface data at the mesoscale complements with that acquired at higher resolution scale by AFM and VSI/PSI in shorter experimental runs, as well as with that from long batch and flow-through experiments, which do not provide direct information on the occurring mineral surface mechanisms.

A promising future perspective involves the integration of a micro-Raman spectrometer to the LCM-DIM setup to provide simultaneous acquirement of the surface topography and chemistry during mineral (phyllosilicate) weathering.

## Experimental

### In situ flow-through experiments

**Figure 6:**
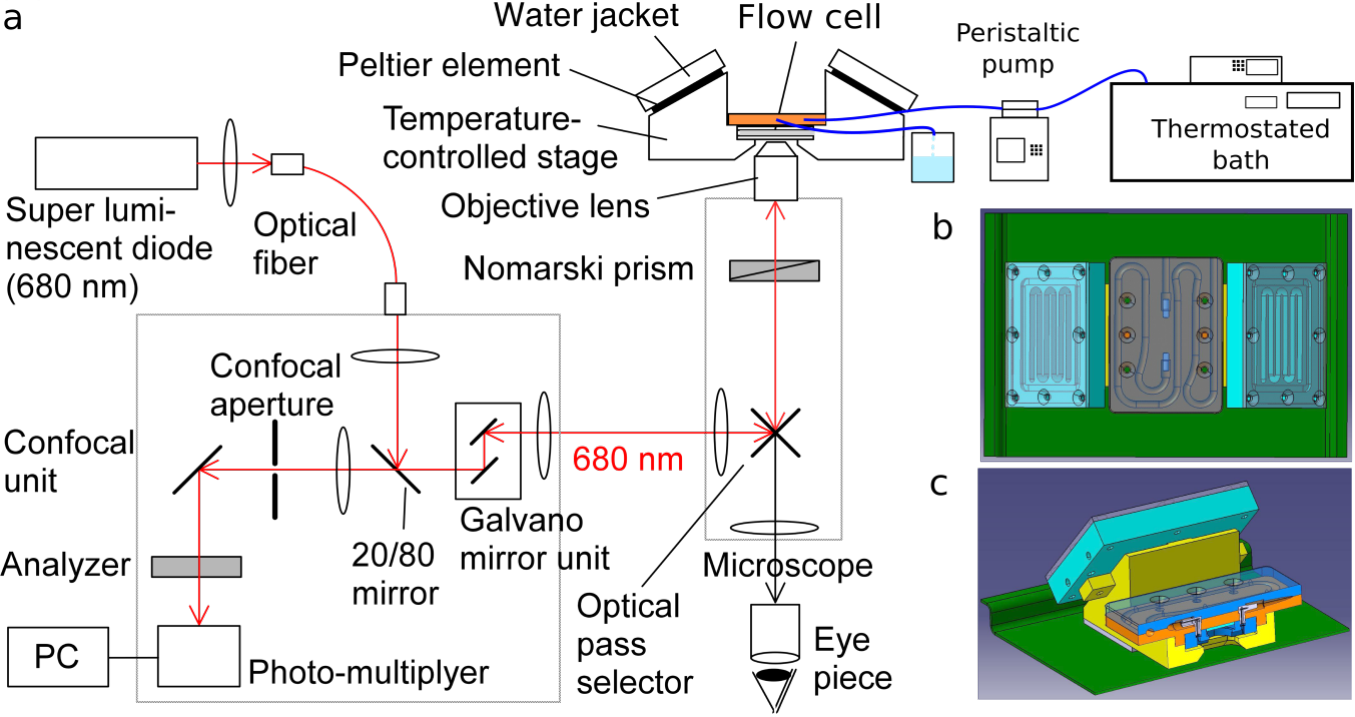
Schematic representation of the experimental setup: (a) Laser confocal differential intereference contrast microscope, (b) top view, and (c) a cross-sectional view of the temperature-controlled observation flow cell.

Changes of the biotite (001) cleavage surface topography were monitored in situ by employing laser confocal microscopy with differential interference contrast microscopy (LCM-DIM, Figure 6a). This advanced optical system is a combination of two microscopy techniques: a confocal system (FV300, Olympus) is attached to an inverted optical microscope (IX70, Olympus) and a Nomarski prism is introduced into the optical path. A curve-matched thermistor and two Peltier elements were employed to precisely control the temperature of the flow-through observation cell (Figure 6b,c). A detailed description of this experimental setup can be found in previous publications [38,51].

The biotite sample used in the present work was from Bancroft-Ontario, Canada and was purchased from Ward’s Natural Science Establishment. Its composition was reported by Turpault and Trotignon [43]. Biotite flakes with (001) cleavage surfaces of ca. 2 × 8 mm^2^ and between 0.08 and 0.15 mm in thickness reacted with solutions of pH 1 (0.1 mol·L^−1^ HNO_3_ and 0.01 mol·L^−1^ NaNO_3_) at 11 and 25 °C and pH 9.5 (0.01 mol·L^−1^ Na_2_B_4_O_7_·10H_2_O and 0.022 mol·L^−1^ NaOH) at 50 °C. All solutions were prepared from ultrapure grade chemicals.

The biotite flakes were fixed parallel to the (001) surface on the bottom of the fissure of a custom-made Teflon flow-through cell by a silicone adhesive. The flow cell was a rectangular prism with a volume of 0.08 cm^3^ (Figure 6c). A small channel on each side of the cell allowed the reacting solution to circulate at a constant flow rate (0.03–0.07 mL·min^−1^), yielding a residence time of approximately 3 to 8 min. The Teflon reactor was carefully sealed with a cover glass glued with high vacuum grease (Dow Corning). The duration of the experiments varied from 2 h to 3 d according to the experimental conditions. Images of the (001) cleavage surface were taken every 20 s to 15 min with a capture time of 9.6 s.

### Solution analysis

The chemical composition of the input and output solutions of the basic pH experiment was determined. Si concentration was determined by colorimetry, using the molybdate blue method [52] with a UV–vis spectrophotometer (Perkin Elmer Lambda 25). The detection limit was 5 ppb and the uncertainty was less than 3%. Al concentration was measured by fluorimetry using lumogallion as complexing agent [53] with a FluoDia T70 high-temperature fluorescence microplate reader fluorimeter. The detection limit was 2 ppb and the uncertainty was less than 5%. Mg concentration was determined by ion chromatography using a Methrohm 883 Basic IC plus with a Metrosep C3 column. The detection limit and the uncertainty were 0.5 ppb and 3%, respectively. Fe concentration was determined by colorimetry, measuring the absorption of the red complex that Fe(II) forms with 1,10-phenanthroline [54–55]. The detection limit was 0.2 ppm and the uncertainty was less than 3%. The pH value was measured by using Crison combination electrodes, calibrated with pH 2, 7 and 9.2 buffer solutions (accuracy ±0.02 pH units).

#### Ex situ sample characterization

Raman spectroscopy coupled to a confocal microscope was used to examine ex situ the chemical composition of the reacted biotite (001) surface to identify possible newly formed phase(s). A lab-RAM spectrometer with backscattering geometry was employed to collect the spectra. A diode laser (λ = 525 nm) exited the surface and the emitted waves were detected with a Peltier cooled charge-coupled device (CCD) (1064 × 256 pixel) [56]. Signal averaging of three spectra was performed with a time acquisition of 300 s.

#### Solution saturation state

Table 2 shows the saturation indexes (SI) of the output solution of the experiment run at pH 9.5 and 50 °C collected between 48 h and the end of the experiment (about 69 h) that were calculated by using the PHREEQC code and the thermodynamic data base LLNL [57]:


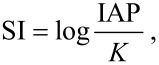


where *K* is the equilibrium constant for the mineral dissolution reaction and IAP is the corresponding ion activity product [43]. The concentration of NO_3_^−^ and Na^+^ was fixed to be 0.01 M and 0.04 M, respectively. Due to the high sodium concentration with respect to potassium, the K^+^ concentration could not be measured due to overlapping cation peaks. Therefore, K^+^ concentration was assumed to be stoichiometric with respect to Si released.
